# Characterization of the juvenile green turtle (*Chelonia mydas*) microbiome throughout an ontogenetic shift from pelagic to neritic habitats

**DOI:** 10.1371/journal.pone.0177642

**Published:** 2017-05-11

**Authors:** James T. Price, Frank V. Paladino, Margaret M. Lamont, Blair E. Witherington, Scott T. Bates, Tanya Soule

**Affiliations:** 1 Department of Biology, Indiana University-Purdue University, Fort Wayne, Indiana, United States of America; 2 U.S. Geological Survey, Gainesville, Florida, United States of America; 3 Disney’s Animals, Science, and Environment, Lake Buena Vista, Florida, United States of America; 4 Department of Biological Sciences, Purdue University Northwest, Westville, Indiana, United States of America; Pusan National University, REPUBLIC OF KOREA

## Abstract

The gut microbiome of herbivorous animals consists of organisms that efficiently digest the structural carbohydrates of ingested plant material. Green turtles (*Chelonia mydas*) provide an interesting model of change in these microbial communities because they undergo a pronounced shift from a surface-pelagic distribution and omnivorous diet to a neritic distribution and herbivorous diet. As an alternative to direct sampling of the gut, we investigated the cloacal microbiomes of juvenile green turtles before and after recruitment to neritic waters to observe any changes in their microbial community structure. Cloacal swabs were taken from individual turtles for analysis of the 16S rRNA gene sequences using Illumina sequencing. One fecal sample was also obtained, allowing for a preliminary comparison with the bacterial community of the cloaca. We found significant variation in the juvenile green turtle bacterial communities between pelagic and neritic habitats, suggesting that environmental and dietary factors support different bacterial communities in green turtles from these habitats. This is the first study to characterize the cloacal microbiome of green turtles in the context of their ontogenetic shifts, which could provide valuable insight into the origins of their gut bacteria and how the microbial community supports their shift to herbivory.

## Introduction

Animal microbiomes are increasingly regarded as vital systems that are key to the host’s daily survival [1–3]. For example, microbes in the animal gut are particularly important as they can aid the host’s immune system, synthesize essential amino acids, and assist in the digestion of complex carbohydrates [3–5]. The characterization of core microbiome constituents and documenting shifts that may occur within these microbial communities, therefore, may be important for gaining insights into animal health or biology [6]. Initiation and development of the gut flora as animals mature can be stimulated by interactions with parents, adults of the same species, or environmental sources [7]. However, these source factors vary among different types of animals; for example, mammals typically have extensive postnatal contact with their mother, which may serve to inoculate the juvenile animal with a developed gut flora from the adult. Reptiles on the other hand often lack maternal contact, thus diminishing the influence from this potential source of microbes. Some reptiles compensate for this lack of exposure to the adult gut flora by directly ingesting feces from adults in their species group or soil from areas surrounding their nest in order to jump-start a healthy gut microflora [8].

As with other reptiles, the green sea turtle (*Chelonia mydas*) lacks maternal care post-oviposition. After their eggs hatch on sandy beaches, *C*. *mydas* hatchlings move offshore where they remain within a surface-pelagic habitat, feeding primarily on animal material such as hydroids and bryozoans, as well as *Sargassum*, a floating brown algae that converges to form a critical developmental habitat [9,10]. After three to six years offshore the turtles move to neritic habitats, such as the bays that provide a refuge for sea grasses and tunicates, both of which are known to be important dietary components for developing green turtles [11]. Throughout this period of early development, juvenile turtles typically have no close contact with adults of their own species, thus environmental and dietary factors likely have an important influence on their developing gut microflora. Green turtles are hindgut fermenters, and as the juveniles move from the pelagic to neritic habitats they gradually become herbivorous [4,12], and thus, we hypothesize that these dietary shifts likely correspond to a marked transition in their gut flora.

To characterize the microbiome of the green sea turtle through this ontogenetic shift, the objectives of this study were to (1) make a novel observation of what bacteria are present within the juvenile green turtle, and (2) compare the bacterial communities of juvenile green turtles before and after the shift from oceanic surface-pelagic habitats to inshore bays populated with sea grasses.

## Materials and methods

### Study sites

**Fig 1:**
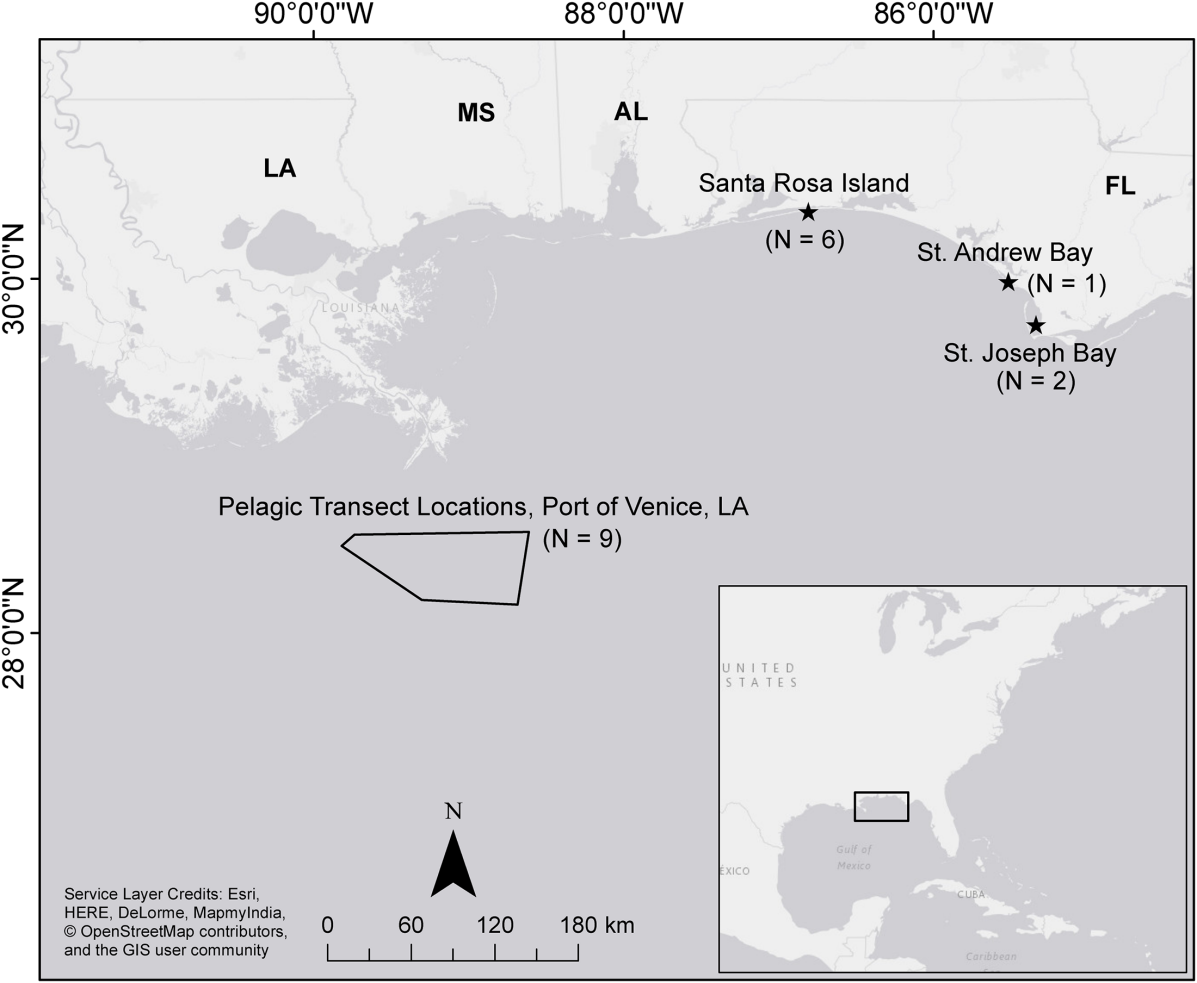
Sampling locations for juvenile green turtles in the Northern Gulf of Mexico (USA). Pelagic transects are bounded by a black polygon and neritic sampling sites are marked by black stars. Inset map of the eastern United States and Gulf of Mexico.

Sampling of juvenile green turtles occurred within pelagic and neritic portions of the Northern Gulf of Mexico (Fig 1). Searches for offshore pelagic-stage green turtles were conducted daily between the 13-18^th^ of July 2015 from the port of Venice, LA, USA. Searches were focused on floating mats of pelagic *Sargassum*, which support communities of rafting invertebrates, zooplankton, fish, and some epiphytic organisms.

Searches for neritic juvenile green turtles were conducted at multiple inshore locations in Northwestern Florida, USA. The initial sampling of green turtles took place in the coastal bays of St. Joseph Bay (SJB) and Crooked Island Sound of St. Andrews Bay (SAB) between the months of September and October 2015. SJB is 21 km in length and 8 km at the widest point, while the study site in SAB has a length of approximately 16 km and a width of 1.6 km. The bays are both populated by large beds of turtle grass (*Thalassia testudinium*), as well as other sea grass species. Sampling of neritic juvenile green turtles was also conducted in a beachfront habitat during October 2015 along 18 km of beach on Santa Rosa Island (SRI), FL, USA owned by Eglin Air Force Base (AFB). Permission for sampling on SRI was granted by Eglin AFB, but no special permissions were required for sampling in SJB, SAB, or any of the offshore sampling in the Northern Gulf of Mexico.

### Sample collection

For each captured juvenile turtle body size was determined, measured as straight carapace length (SCL), from the nuchal notch at the anterior edge of the carapace to the posterior tip of the supracaudal scutes. Following measurement, cloacal swabs were taken. Although cloacal swabs are often used as a proxy for fecal samples to characterize the animal’s gut flora [13], swabs from the turtles sampled for this study are likely influenced by the surrounding environment due to the turtle’s constant contact with water in the marine setting. To minimize this environmental influence and the potential for contamination from surrounding skin microflora, the exterior edge of the cloaca and tail of each turtle were sterilized with a 70% isopropyl alcohol swab after being placed on a table (wiped with 70% isopropyl alcohol) ventral side-up. Swabbing was carried out with sterile ultrafine (smaller pelagic turtles) or standard (larger neritic turtles) Hydraflock^®^ swabs (Puritan Medical, Guilford, Maine, USA). The swabs were dipped into sterile water before being inserted approximately the length of the swab tip (~ 2 cm) into the cloaca and rotated 2–3 times before removal. The swab tip was then cut from the handle using sterilized scissors and stored in RNAlater^®^ solution (ThermoFisher Scientific, Waltham, Massachusetts, USA), which allows extended storage at temperatures above -20°C. Duplicate swabs were taken from each turtle, and then all swab samples were stored at ~4°C in solution for up to five days before being transferred to -20°C for long term storage until thawing for DNA extraction.

For the pelagic juvenile turtle sampling (N = 9), capture methods followed those previously described [9]. During time onboard (approximately 1-hour), captured turtles were kept on a sterilized surface for opportunistic collection of fecal samples; however, only one individual produced a fecal sample. This sample was collected aseptically in a sterile Eppendorf tube containing RNAlater^®^ and stored as described above. Capture techniques for neritic juveniles from the bays (N = 3) and beachfront (N = 6) were location-dependent. In SJB and SAB, the capture methods were followed as previously reported [14]. Due to poor weather conditions and marine mammal sightings, our ability to consistently use the tangle net was limited and therefore one turtle was caught opportunistically with a dip net following a sighting in SJB. Sampling in the beachfront habitat on SRI used both utility task and all-terrain vehicles, which were driven along the beach while observers looked for juvenile green turtles in the surf zone. If a turtle was spotted, a 10 m tangle net was used to seine in front of the individual, while the remaining team members approached from behind the turtle to coerce it into the tangle net using dip nets. Five individuals were captured on SRI, and one individual was recaptured one week later for a second sample.

All animal sampling was carried out in strict accordance with the Purdue Animal Care and Use Committee (PACUC) policies and guidelines, and under PACUC approved protocol #1502001194. All captured turtles were released shortly after sampling was complete.

### Bacterial DNA analysis

Cloacal swabs and the fecal sample were processed using the MoBio PowerSoil^®^ DNA Isolation Kit (MoBio Laboratories, Carlsbad, California, USA). The manufacturer’s protocol for low biomass samples was followed throughout the DNA extraction with slight modifications to maximize yield [15]. Adjustments included (1) incubating samples in the PowerBead solution at 65°C for 15 minutes before vortexing with the PowerBead tubes and (2) using only 30 μL of the elution agent (Solution C6) two separate times for a total elution volume of 60 μL, instead of the recommended 50 μL, before subsequently allowing it to remain on the filter membrane for up to 10 minutes for each elution.

The 16S rRNA gene was amplified through a PCR and next-generation “targeted-amplicon sequencing” approach [16] using the bacterial primers, CS1_515F (5’ ACACTGACGACATGGTTCTACA-GTGCCAGCMGCCGCGGTAA 3’) and CS2_806R (5’ TACGGTAGCAGAGACTTGGTCT-GGACTACHVGGGTWTCTAAT 3’). These primers, which targeted the V4 region of the 16S rRNA, contained CS1 and CS2 linkers to allow for subsequent application of adapter sequences and sample-specific barcodes [17]. Sequence amplification was completed in 20 μL reactions with a final concentration of 500 nM for each primer, 2–9.8 μL of template DNA (dependent upon how much was required for successful amplification), and sterile water. The PCR thermal cycling conditions were as follows: 5-minute denaturation period at 95°C, followed by 28 cycles of 95°C for 30 seconds, 55°C for 45 seconds, and 68°C for 30 seconds. A final 7-minute period of elongation was performed at 68°C. For the second stage of the two-stage PCR process, we processed the amplicons using previously described methods [16], with a final concentration of 400 nM for each primer with cycling conditions as follows: 95°C for 5 minutes, followed by 8 cycles of 95°C for 30 seconds, 60°C for 30 seconds, and 68°C for 30 seconds. A final elongation period was performed at 68°C for 7 minutes. These amplicons were subsequently prepared for multiplexed sequencing on an Illumina MiSeq sequencer [16,18]. The second stage of the PCR process and the Illumina sequencing were completed by the DNA Services Facility at the University of Illinois at Chicago.

Illumina sequencing provided an output of paired-end reads (2 x 250 base pairs) for each sample. The reads were paired using the PEAR software package 0.9.6 [19] and then the FASTQ output files were imported to the CLC Genomics Workbench software package (CLC Bio, Aarhus, Denmark) for quality filtering and length trimming (anything with <250 base pairs or a quality score <20 was discarded). Following this, the usearch61 algorithm was used to remove chimeras and the QIIME software package version 1.9.0 [20] was used for all subsequent sequence analyses. The sequences were deposited in the Sequence Read Archive database of NCBI (Accession no. SRP080996).

After the initial processing, the sequences were clustered into operational taxonomic units (OTUs) based on a 97% similarity threshold using the denovo UCLUST algorithm [21]. A representative sequence from each OTU was used to assign taxonomic classifications using the 16S rRNA Greengenes 13_8 database as a reference [22]. Sequences observed below a frequency of 0.005% among all samples were subsequently filtered to reduce the possibility of including sequencing errors [23]. For comparisons of communities from cloacal swabs, the samples were rarified to an even depth of 63,000 sequences for alpha and beta diversity metrics. Alpha diversity was measured as observed taxa (richness), equitability, and Shannon-Weiner diversity. Beta diversity comparisons were visualized using unweighted and weighted non-metric multidimensional scaling (NMDS) plots calculated using the Bray-Curtis dissimilarity. For these comparisons of diversity, the samples were categorized only by habitat type since the body size and putative diet of the turtles was consistent within each of the habitats.

### Statistical analysis

All statistical analyses were completed using the R software package version 3.2.3 (www.r-project.org). Due to the disparate sample size of individuals from each habitat type, a Kruskal-Wallis analysis of variance was used to compare the body sizes of juvenile green turtles between habitats, followed by a Dunn’s test for post hoc analysis. To evaluate the diversity of bacterial communities between turtles from each habitat type, an analysis of similarities (ANOSIM) was performed on the datasets produced by each beta-diversity algorithm to assess the statistical significance (p-value) of each comparison. An ANOSIM also produces an R^2^ value between 0 and 1, where a higher value represents a more dissimilar relationship between the bacterial communities.

## Results

### Illumina sequencing output

A total of 1,642,272 quality reads with an average length of 295 bp were generated from 19 samples (18 cloacal swabs and one fecal sample). The samples had a median of 84,378 reads, which clustered into 792 unique OTUs. The bacterial communities largely consisted of three phyla (~94% of all sequences), the Proteobacteria, Bacteroidetes, and Firmicutes, however the taxa within these phyla varied widely among individuals.

### Body size

To determine if variation between bacterial communities was related to the size of the green turtles, the body size of all individuals was measured as straight carapace length (SCL) and was compared between habitat types. Pelagic juvenile green turtles (N = 9) had a mean SCL of 19.0 ± 1.49 cm. The SCL for individuals in neritic areas varied somewhat by habitat type; as turtles from the beachfront (N = 6) had a mean body size of 31.4 ± 1.80 cm, while turtles from the bays (N = 3) had a mean body size of 41 ± 5.48 cm. The body sizes of turtles in the pelagic habitat differed significantly from turtles in the beachfront (p = 0.004) and bay (p < 0.001) habitats. The turtles from the beachfront and bay habitats did not have significantly different body sizes (p > 0.05).

### Diversity of bacterial communities

Alpha diversity of cloacal microbial communities was calculated to assess variations within each habitat type. There were no significant differences in alpha diversity (observed OTUs, evenness, and Shannon-Weiner diversity index) in the cloacal bacterial communities of juvenile green turtles from pelagic and neritic habitats (p > 0.05, data not shown). When neritic sampling sites were divided into beachfront and bay habitats (Table 1), the mean observed OTUs in cloacal samples from each site did have greater variation, but no significant differences were detected (p > 0.05).

**Table 1:** Alpha diversity of bacterial communities in juvenile green turtle cloacal samples^a^^,^^b^.

*Habitat*	*Observed OTUs*	*Equitability*	*Shannon-Weiner Diversity*
Pelagic	375.2 ± 33.8	0.600 ± 0.065	5.13 ± 0.60
Beachfront	394.8 ± 57.0	0.572 ± 0.046	4.92 ± 0.45
Bay	377.0 ± 112.5	0.580 ± 0.104	4.94 ± 0.97

^a^Error is represented by ± 1 SD

^b^No significant differences (p > 0.05) were detected among the alpha diversity metrics

**Fig 2:**
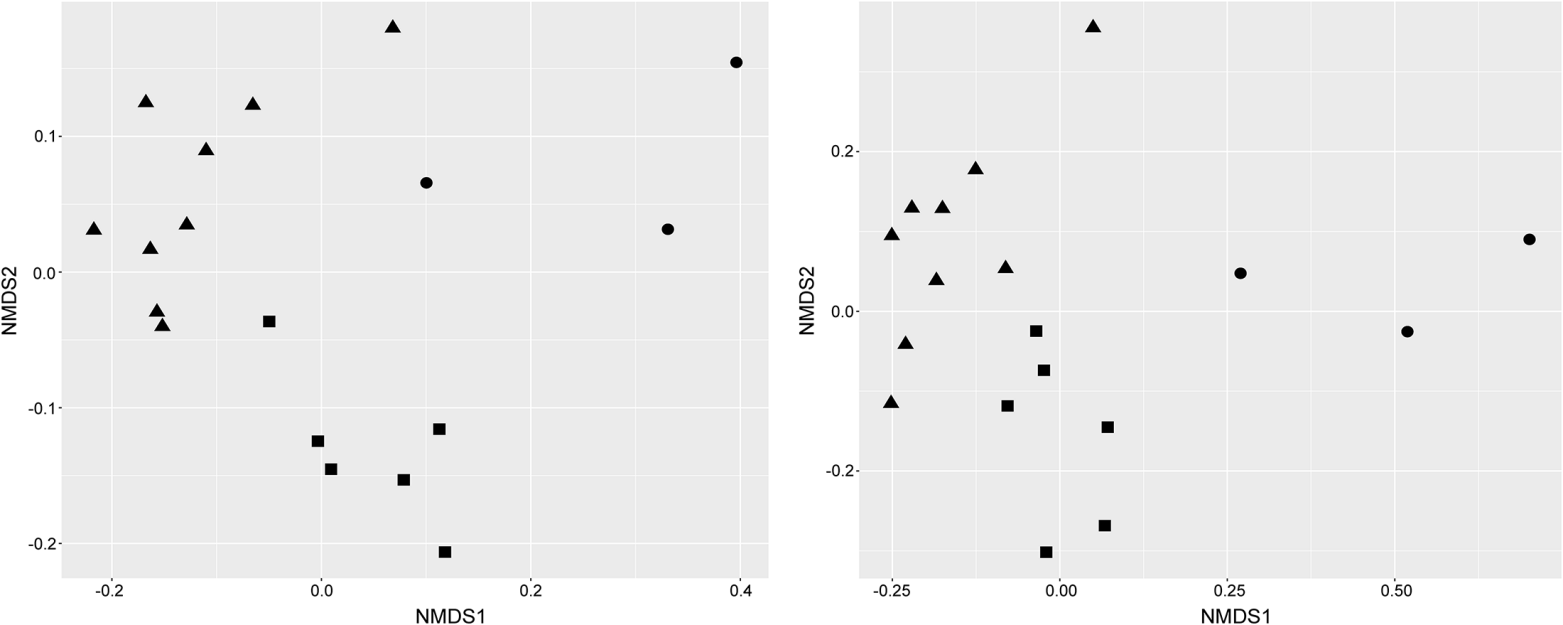
NMDS plot of bacterial community similarity between cloacal samples with significant clustering (p < 0.05) by each habitat type. Cloacal samples from juvenile green turtles are differentiated by habitat type: pelagic (triangle), beachfront (square), and bay (circle) habitat. Plots were generated using an (A) unweighted (transformed to a distance matrix considering only presence/absence), and (B) weighted (not transformed) Bray-Curtis dissimilarity.

**Fig 3:**
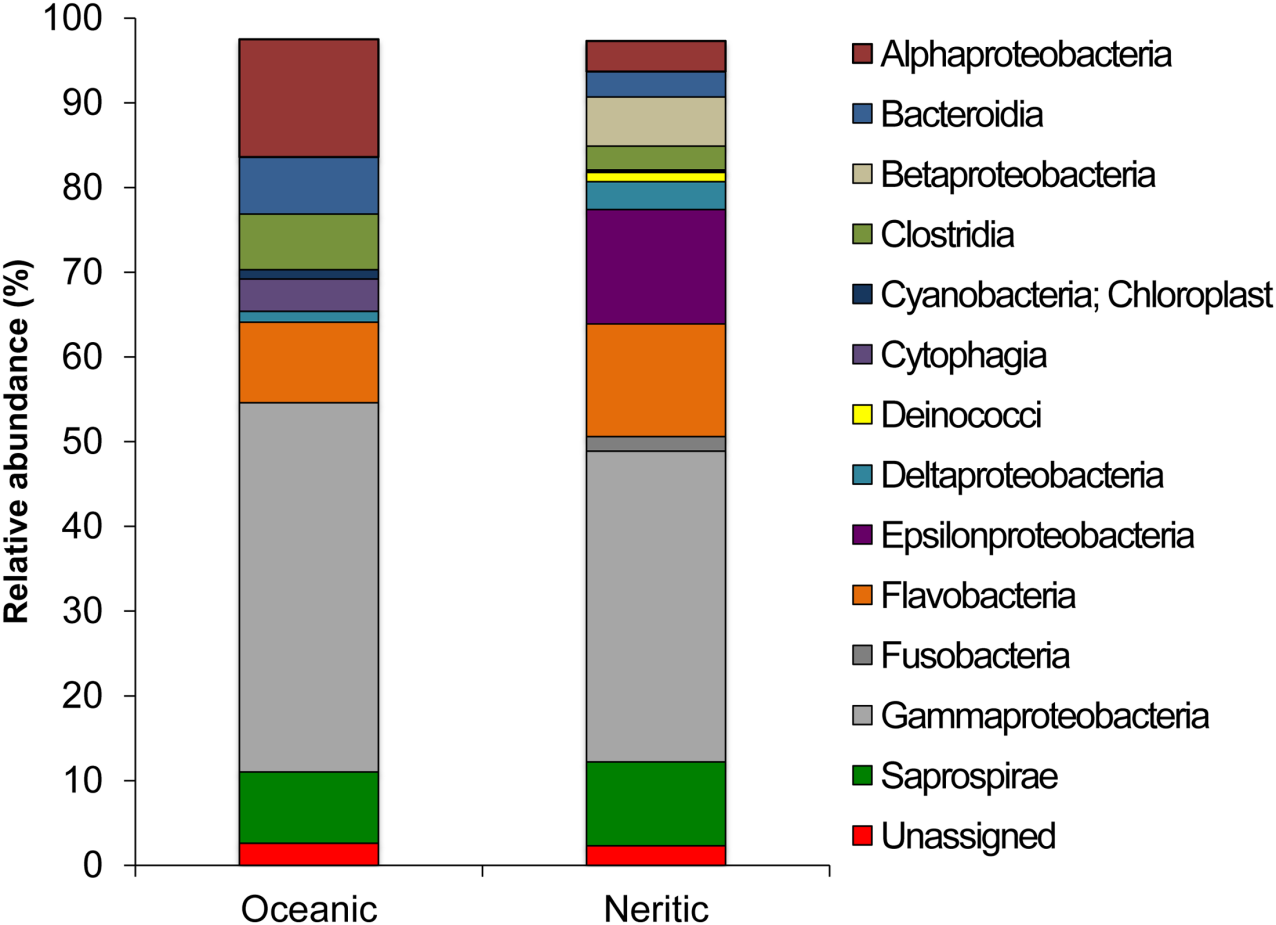
Bacterial class community composition of cloacal swabs from juvenile green turtles. Sampled individuals were categorized by habitat type.

Beta diversity, the variation of cloacal microbial communities between habitats, was calculated using Jaccard’s index and the Bray-Curtis dissimilarity. Beta diversity varied significantly between cloacal bacterial communities of turtles from pelagic and neritic environments (Table 2). Similarly, when samples from turtles in pelagic habitats were compared to those from the two neritic habitat types, the three sample groups were significantly different. Bacterial communities of turtles captured from the beachfront were unique, but were more similar to the pelagic turtles than the bay turtles (Fig 2, Table 2). Bacterial communities of cloacal samples from all turtles were largely represented by 23 taxa across the three previously noted phyla (Proteobacteria, Bacteroidetes, and Firmicutes) that had a relative abundance above 2% in cloacal samples from at least one habitat type (Fig 3, Table 3).

**Table 2:** Comparison of bacterial communities in juvenile green turtle cloacal samples^a^^,^^b^.

*Groups*	*N*	Jaccard's Index		Bray-Curtis Dissimilarity	
		*R*^*2*^	*p-value*	*R*^*2*^	*p-value*
Pelagic vs. Neritic	18	0.562	0.001***	0.437	0.001***
Pelagic vs. Beachfront	15	0.767	0.002**	0.545	0.001***
Pelagic vs. Bay	12	0.854	0.004**	0.939	0.005**
Beachfront vs. Bay	9	0.901	0.014*	0.988	0.010**

^a^Distance metrics were compared using an analysis of similarities (ANOSIM) with 999 permutations

^b^Significance levels: p ≤ 0.05*,

p ≤ 0.01**,

p ≤ 0.001***

**Table 3:** Relative abundance of bacterial taxa in juvenile green turtle cloacal samples.

*Bacterial Taxa*^a^	*Pelagic*	*Beachfront*	*Bay*
**Phylum Bacteroidetes**			
Order Bacteroidales; Unclassified	3.0%	0.5%	2.1%
Genus *Bacteroides*	2.0%	0.1%	2.3%
Order Cytophagales; Genus *Microscilla*	2.7%	ND	ND
Family Flavobacteriaceae; Unclassified	3.9%	4.4%	1.5%
Genus *Tenacibaculum*	2.0%	5.1%	0.9%
Family Saprospiraceae; Unclassified	7.0%	13.8%	0.7%
**Phylum Firmicutes**			
Order Clostridiales; Unclassified	3.9%	0.2%	0.4%
Family Lachnospiraceae; Unclassified	0.6%	ND	3.2%
Genus *Fusibacter*	0.1%	ND	2.2%
**Phylum Proteobacteria**			
Family Hyphomicrobiaceae; Unclassified	4.2%	0.2%	ND
Family Rhodobacteraceae; Unclassified	8.3%	4.0%	1.6%
Family Neisseriaceae; Unclassified	0.1%	ND	16.1%
Family Desulfobulbaceae; Unclassified	ND	ND	8.4%
Family Campylobacteraceae; Unclassified	0.1%	ND	17.8%
Genus *Arcobacter*	ND	5.5%	5.6%
Genus *Campylobacter*	ND	ND	2.9%
Class Gammaproteobacteria; Unclassified	6.8%	14.9%	0.6%
Order Alteromonadales; Unclassified	2.8%	0.7%	0.2%
Genus *Shewanella*	3.7%	3.1%	ND
Order Cardiobacterales; Unclassified	6.9%	4.6%	3.2%
Family Moraxellaceae; Unclassified	11.7%	1.5%	0.8%
Genus *Moraxella*	3.1%	15.9%	4.4%
Family Pseudoalteromonadaceae; Unclassified	4.2%	4.1%	0.2%

^a^Bacterial groups are classified to the genus or next lowest classification level and only those with a significant relative abundance (>2%) in turtles from at least one habitat type are represented in this table. ND = not detected or <0.01%

While no statistical analyses could be used to compare our single fecal sample to the cloacal sample from the same individual, there were similarities between the bacterial communities observed in these two sample types. The bacterial classes Clostridia (63.0%) and Bacteroidia (31.1%) dominated the single fecal sample, while the cloacal swab from the same individual (WP940) also had high abundances of Bacteroidia (24.5%) and Clostridia (16.5%). Further comparisons of the bacterial communities in each sample are listed in S1 Table.

## Discussion

Overall, members of the Proteobacteria were the most abundant taxa in our cloacal samples across all three habitat types. Gammaproteobacteria in the families Moraxellaceae and Shewanellaceae were well represented in cloacal samples from turtles in the pelagic and beachfront habitats. The Moraxellaceae are known to be common inhabitants of the mucous membranes of marine animals and have the potential to be parasitic or assist with digestion within the gastrointestinal tract [24]. The Shewanellaceae are common on algae, such as *Sargassum* in the surface-pelagic zone, and facultative anaerobes within this family have been isolated previously from the gut of other marine animals [25]. A high abundance of Epsilonproteobacteria of the order Campylobacterales were found in turtles from the neritic habitats. Campylobacterales are common components of the gut microflora of animals and have been previously documented as members of the gut community in loggerhead sea turtles (*Caretta caretta*), a largely carnivorous species [26]. However, it is unlikely they are directly associated with a shift towards a plant-based diet in neritic turtles since these bacteria are not known to ferment complex carbohydrates [27]. It is also possible that the presence of Campylobacterales in these samples is attributable to the proximity of our neritic sites to the coast, as near-shore accumulation of Campylobacterales due to runoff from terrestrial watersheds has been previously documented in northwestern Florida [28].

Bacteroidetes and Firmicutes were also quite abundant, cumulatively accounting for approximately 30% of the bacteria in our cloacal samples across the three habitat types. Bacteroidetes were found in all samples, primarily among the genera *Bacteroides*, *Paludibacter*, and *Tenacibaculum*. The genus *Bacteroides* contains bacteria that are often saccharolytic members of the gut microflora and are among the most common clinically isolated anaerobe [29], while the *Paludibacter* and *Tenacibaculum* are often associated with marine habitats and animals [30,31]. Firmicutes, which were only common in the pelagic and bay habitats, were represented almost entirely by bacteria from the class Clostridia. Within Clostridia, the family Lachnospiraceae were more dominant in the turtles from bay habitats, while the Ruminococcaceae were more common in those from the pelagic habitat. Both families are common gut microflora of animals where they are known to metabolize complex carbohydrates like cellulose [32]. It is possible that members of these different families specialize in breaking down specific dietary components as turtles shift from the animal and *Sargassum*-based diet in the pelagic habitat toward one that is primarily seagrass in the bay.

Relative abundance of the Firmicutes, however, was lower than expected in comparison with other studies of the reptilian gut microbiome [26,33,34]. Recent work by Abdelrhman et al., which to our knowledge is currently the only published culture-independent study to characterize gut microbial communities of live sea turtles, found much higher abundances of Firmicutes within captive loggerhead turtles [26]. One likely reason for this disparity is that the cloacal microbiome is simply different than the intestinal microbiome in reptiles, due to factors such as environmental influence and aerobic conditions. Similar to our findings, some studies of the cloacal and fecal microbiome in reptiles found higher relative abundances of Proteobacteria [35] and Bacteroidetes [36] than Firmicutes. This suggests that the low relative abundance of Firmicutes in the green turtle cloaca resembles what has been observed in other reptiles and that the cloacal microbiome may be more influenced by habitat than the gut.

Interestingly, cloacal bacterial communities of juvenile green turtles sampled for this study had a consistent level of alpha diversity and evenness between habitats. This could indicate that the complexity of the microbial communities in juvenile turtles is established before the ontogenetic stages sampled in this study [37,38]. Comparisons of beta diversity, however, exhibited significant differences in the clustering of bacterial communities between pelagic and neritic juvenile green turtles (Table 2). Although two different algorithms (Jaccard’s index and Bray-Curtis dissimilarity) were used to compare these bacterial communities, both metrics clearly demonstrate important microbial community shifts occur as these turtles’ transition from one habitat type to another. These changes in community structure are likely predominantly due to environmental differences, but may be secondarily attributed to other factors, such as different dietary resource use.

The beta diversity metrics also exhibited significant differences between the bacterial communities of neritic turtles sampled from beachfront and bay habitats (See Table 2). Indeed, bacterial communities of turtles within the beachfront habitat were more similar to those from pelagic habitats than from the bays. This may be due to the pelagic and beachfront habitats being open marine systems, rather than being generally enclosed, as in the bays. Green turtles found in bay habitats had a mean body size near 41 cm (SCL), while those residing near the beachfront were approximately 10 cm smaller, on average. The generally smaller body size of the beachfront turtles relative to those from the bays, coupled with the variation in bacterial communities, may indicate that the juveniles using the two neritic habitat types are in different developmental stages. This result supports the notion that the beachfront habitat of Santa Rosa Island may serve as a distinct habitat for green turtles at an intermediate developmental stage that are in transition from the pelagic to bay habitats [37].

The cloacal swabs served as reasonable proxies for fecal samples, which were difficult to obtain from active turtles in this marine habitat. When compared with the fecal sample from the same individual, the cloacal sample included similar bacterial groups, such as Bacteroidia, Clostridia, and Deltaproteobacteria. However, the cloacal sample from this individual was more diverse and included some groups not found in the fecal samples, such as the Gammaproteobacteria. Before a complete comparison can be made between cloacal and fecal samples, however, a larger sample size will be necessary.

In conclusion, as juvenile green turtles encounter different dietary resources during their recruitment from an offshore surface-pelagic habitat towards neritic developmental foraging areas, significant changes in the bacterial communities of their cloaca can be discerned. While some differences between the cloacal and fecal samples were observed, variation in cloacal bacteria between habitat types reflects clear changes in the gut microflora of the juvenile turtles. Future studies examining the gut microflora more directly through targeted analysis of the microbial community of the gastrointestinal tract through fecal material or gastric lavage samples would serve to clarify this issue. As the turtle gut flora are critical for proper digestion and can contribute essential nutrients as well as immune support, these microbes have the potential to directly influence sea turtle health, thus future work focusing on the fundamental effects that bacterial communities have on a turtle’s diet, development, and health is needed. It is particularly important to focus on young turtles, including hatchlings and post-hatchlings, stages not sampled in this study, as they are often more sensitive to disease and short term environmental changes, such as temperature and pollution. Thus, microbiome data from within this population could also provide information with relevance for successful rehabilitation and protection programs [38]. Overall, our study represents a first step toward elucidating bacterial communities’ responses to shifts in habitat and diet in developing sea turtles, though other groups that may also represent healthy turtle gut flora, such as archaea and fungi, remain poorly characterized.

## Supporting information

S1 Table Relative abundance of bacterial taxa in a juvenile green turtle fecal and cloacal sample^a^. (DOCX)
