# Supplementary material for: Characterization of the juvenile green turtle (Chelonia mydas) microbiome throughout an ontogenetic shift from pelagic to neritic habitats
Source: PLoS One. 2017 May 11;12(5):e0177642. doi: 10.1371/journal.pone.0177642 (PMC5426784; doi:10.1371/journal.pone.0177642)
Supplement: S1 Table — (DOCX) [file pone.0177642.s001.docx]

| **S1 Table** Relative abundance of bacterial taxa in a juvenile green turtle fecal and cloacal sample^a^ | | |
| --- | --- | --- |
| *Bacterial Taxa^b^* | *Fecal* | *Cloacal* |
| **Phylum Bacteroidetes** |  |  |
| Order Bacteroidales; Unclassified | 27.3% | 21.9% |
| Family Rikenellaceae; Unclassified | 3.1% | 1.9% |
| Family Saprospiraceae; Unclassified | ND | 9.0% |
| **Phylum Firmicutes** |  |  |
| Order Clostridiales; Unclassified | 25.7% | 13.2% |
| Family Lachnospiraceae; Unclassified | 7.6% | 0.6% |
| Genus *Coprococcus* | 6.3% | 0.1% |
| Genus *Epulopiscium* | 18.4% | 0.5% |
| Family Ruminococcaceae; Unclassified | 2.9% | 1.1% |
| **Phylum Proteobacteria** |  |  |
| Family Rhodobacteraceae; Unclassified | ND | 6.8% |
| Family Desulfovibrionaceae; Unclassified | 3.1% | 1.4% |
| Class Gammaproteobacteria; Unclassified | ND | 15.7% |
| Order Cardiobacterales; Unclassified | ND | 5.3% |
| Family Moraxellaceae; Unclassified | ND | 2.4% |
| Family Xanthomonadaceae; Unclassified | ND | 2.7% |
| ^a^The individual turtle (WP940) represented in this table was captured in the pelagic habitat ^b^Bacterial groups are classified to the genus or next lowest classification level and only those with a significant relative abundance (>2%) in at least one sample type are represented in this table. ND = not detected or <0.01% | | |
|  | | |
